# The Role of Microglia in Retinal Neurodegeneration: Alzheimer's Disease, Parkinson, and Glaucoma

**DOI:** 10.3389/fnagi.2017.00214

**Published:** 2017-07-06

**Authors:** Ana I. Ramirez, Rosa de Hoz, Elena Salobrar-Garcia, Juan J. Salazar, Blanca Rojas, Daniel Ajoy, Inés López-Cuenca, Pilar Rojas, Alberto Triviño, José M. Ramírez

**Affiliations:** ^1^Instituto de Investigaciones Oftalmológicas Ramón Castroviejo. Universidad Complutense de MadridMadrid, Spain; ^2^Departamento de Oftalmología y ORL, Facultad de Óptica y Optometría, Universidad Complutense de Madrid (UCM)Madrid, Spain; ^3^Departamento de Oftalmología y ORL, Facultad de Medicina, Universidad Complutense de Madrid (UCM)Madrid, Spain; ^4^Servicio de Oftalmología, Hospital Gregorio MarañónMadrid, Spain

**Keywords:** microglia, neuroinflammation, Alzheimer's Disease, Parkinson, glaucoma, retina, beta-amyloid, synuclein

## Abstract

Microglia, the immunocompetent cells of the central nervous system (CNS), act as neuropathology sensors and are neuroprotective under physiological conditions. Microglia react to injury and degeneration with immune-phenotypic and morphological changes, proliferation, migration, and inflammatory cytokine production. An uncontrolled microglial response secondary to sustained CNS damage can put neuronal survival at risk due to excessive inflammation. A neuroinflammatory response is considered among the etiological factors of the major aged-related neurodegenerative diseases of the CNS, and microglial cells are key players in these neurodegenerative lesions. The retina is an extension of the brain and therefore the inflammatory response in the brain can occur in the retina. The brain and retina are affected in several neurodegenerative diseases, including Alzheimer's disease (AD), Parkinson's disease (PD), and glaucoma. AD is an age-related neurodegeneration of the CNS characterized by neuronal and synaptic loss in the cerebral cortex, resulting in cognitive deficit and dementia. The extracellular deposits of beta-amyloid (Aβ) and intraneuronal accumulations of hyperphosphorylated tau protein (pTau) are the hallmarks of this disease. These deposits are also found in the retina and optic nerve. PD is a neurodegenerative locomotor disorder with the progressive loss of dopaminergic neurons in the substantia nigra. This is accompanied by Lewy body inclusion composed of α-synuclein (α-syn) aggregates. PD also involves retinal dopaminergic cell degeneration. Glaucoma is a multifactorial neurodegenerative disease of the optic nerve, characterized by retinal ganglion cell loss. In this pathology, deposition of Aβ, synuclein, and pTau has also been detected in retina. These neurodegenerative diseases share a common pathogenic mechanism, the neuroinflammation, in which microglia play an important role. Microglial activation has been reported in AD, PD, and glaucoma in relation to protein aggregates and degenerated neurons. The activated microglia can release pro-inflammatory cytokines which can aggravate and propagate neuroinflammation, thereby degenerating neurons and impairing brain as well as retinal function. The aim of the present review is to describe the contribution in retina to microglial-mediated neuroinflammation in AD, PD, and glaucomatous neurodegeneration.

## Introduction

Alzheimer's Disease (AD) and Parkinson's Disease (PD) are the most common neurodegenerative disorders (de Lau and Breteler, 2006). AD involves progressive memory loss and dementia (Sharma and Lipincott, 2017), while the PD is a chronic and progressive movement disorder (Orr et al., 2002). Glaucoma, a neurodegenerative disease of the optic nerve, is characterized by death of retinal ganglion cells (RGCs) (de Hoz et al., 2016). Recently, neurodegenerative lesions have been detected in the intracranial optic nerve, the lateral geniculate nucleus, and the visual cortex (Gupta et al., 2006, 2007), suggesting that this pathology could be grouped as a neurodegenerative disease (Yucel et al., 2003).

AD is a neurodegenerative disorder related to age, in which neuronal and synaptic losses in the cerebral cortex lead to cognitive impairment, behavioral deficits and dementia (Ghiso et al., 2013). The major pathology related to AD is the extracellular deposit of β-amyloid (Aβ) in the form of parenchymal plaques and cerebral amyloid angiopathy co-existing with intraneuronal accumulations of hyperphosphorylated tau (pTau) (neurofribillary tangles) (Ghiso et al., 2013). These deposits can induce neuronal death by apoptosis (Garcia-Ospina et al., 2003). Initially, it was thought that age was the main risk factor for this disease. However, it is now known to have a multifactorial origin and it seems to result from a complex interaction of multiple environmental and genetic factors (Wostyn et al., 2010). AD has been related to genetic mutations, among them in the gene encoding the Aβ precursor protein peptide, mutations in the presenilins genes (Calabrese et al., 2001) and the presence of the APOE ε4 allele (Martínez-Lazcano et al., 2010). In addition, AD is frequently associated with vascular dysfunctions and inflammation (Dudvarski Stankovic et al., 2016).

PD is characterized by the progressive loss of dopaminergic neurons in the substantia nigra pars compacta and the nerve terminals in the striatum (Dauer and Przedborski, 2003). The clinical symptoms of PD are mainly motor problems, including bradykinesia, rigidity, tremors, and postural instability. In addition, PD presents non-motor symptoms including disorganized speech and altered moods (Fakhoury, 2016). The loss of neurons is accompanied by abnormal intracytoplasmic filamentous aggregates called Lewy bodies. These aggregates (deposited in somas and axons) are constituted by α-syn, parkin, phosphorylated neurofilament and components of the protheosomic-ubiquitin pathway (Orr et al., 2002). The main etiological factors proposed for PD are aging, environmental toxins, and genetic factors. Neurodegeneration could be due to exposure to dopaminergic neurotoxins e.g., herbicides (MPTP), insecticides (Rotenone), and metals (Hernández-Montiel, 2006). Genetic factors include mutations in α-syn (Olanow and Tatton, 1999; Pérez and Arancibia, 2007), while mitochondrial dysfunction and oxidative stress may also act by causing the accumulation of misfolded proteins (Dauer and Przedborski, 2003).

Glaucoma is an age-related multifactorial neurodegenerative disease of the optic nerve, with an irreversible decrease in RGCs, causing a visual-field loss and cupping of the optic nerve head (Quigley et al., 1988). In glaucoma, increased intraocular pressure (IOP), vascular dysregulation, and the immune system activation can trigger several changes in retina and optic nerve including: disrupted axonal transport and neurofilament accumulation, microvascular abnormalities, extracellular matrix remodeling, and glial cell activation. These alterations can lead to secondary damage such as, excitotoxicity, neurotrophin deprivation, oxidative damage, mitochondrial dysfunction, and eventually RGC death (Nickells, 1999; Gallego et al., 2012). In addition, there is a dendritic atrophy of the lateral geniculate nucleus, the site to which the RGC axons project (Gupta et al., 2007; Park and Ou, 2013).

The AD, PD and glaucoma share certain biological features, for example: (i) they are slow and chronic neurodegenerative disorders with a strong age-related incidence; (ii) they have similar mechanisms of cell injury and deposition of protein aggregates in specific anatomical areas (Wostyn et al., 2010; Kaarniranta et al., 2011; Ghiso et al., 2013); and (iii) death occurs in one or more populations of neurons (RGCs in glaucoma, hippocampal and cortical neurons in AD and nigrostriatal dopaminergic neurons in PD) (Mattson, 2000). Although the exact mechanism bringing about this neuronal death remains unknown, these neurodegenerative disorders seem to have pathogenic mechanisms in common. These mechanisms include: oxidative stress (Uttara et al., 2009), mitochondrial dysfunction (Lee et al., 2011; Lascaratos et al., 2012; Chrysostomou et al., 2013), alterations in the ubiquitin-proteasome system (Campello et al., 2013), abnormal accumulation of misfolded proteins, glutamate excitotoxicity (Gazulla and Cavero-Nagore, 2006; Guimaraes et al., 2009), and glial activation and inflammation (Verkhratsky et al., 2014; Brown and Vilalta, 2015). These mechanisms could act individually or synergistically (Ghiso et al., 2013).

Inflammation is a defensive process of the body against damage that seeks to restore tissue integrity. Neuroinflammation, the inflammation of central nervous system (CNS), is essential to protect the tissue. However, uncontrolled and prolonged neuroinflammation is potentially harmful and can cause cellular damage. The astrocytes and microglia could play a major role in the neuroinflammation associated with neurodegenerative diseases (Cherry et al., 2014). The presence of reactive astrocytes, the microglial activation, and the release of inflammatory mediators such as cytokines, reactive oxygen species (ROS), nitric oxide (NO), and Tumor Necrosis Factor-α (TNF-α) could cause a state of chronic inflammation that may exert neurotoxic effects (Cuenca et al., 2014).

The neuroinflammatory process occurs not only in the brain, but also in the retina, which is a projection of the CNS. The retina and brain are associated over a range of neurological and neurovascular conditions of varying etiologies, because the retina and brain are similar, and respond similarly to disease. Thus, it has been described that the retina is a “window to the brain,” and the manifestation of disease in the brain is the same as in the retina (MacCormick et al., 2015). The neuroinflammatory changes could be observed using optical coherence tomography (OCT), a routinely diagnostic techniques used in ophthalmology. This technique provides anatomic detail of pathological changes in the retina and optic nerve. Changes in OCT measurements have been used to study the course of particular neurologic diseases such AD (Garcia-Martin et al., 2014; Maldonado et al., 2015; Salobrar-Garcia et al., 2015, 2016a,b), PD (Yu et al., 2014; Stemplewitz et al., 2015; Boeke et al., 2016; Satue et al., 2017), and glaucoma (Leung, 2016; Fallon et al., 2017), suggesting that the data compiled may be useful as a biomarker in diagnosing and treating neurodegenerative disease.

The aforementioned data underline the importance of knowing the function of inflammatory processes in the retina of neurodegenerative diseases (AD, PD, and glaucoma), especially the contribution to microglial-mediated neuroinflammation.

## Microglial activation

In neurodegenerative diseases, neuroinflammation constitutes a fundamental process in which microglial cells play a key role (Glass et al., 2010). Microglial cells are CNS resident immune cells which have sensor and effector functions as well as phagocytic capacity (Streit et al., 2005). In the developing of CNS these cells enter from the bloodstream, develop from monocytes, and differentiate into microglia. Thus, they maintain numerous cellular antigens present in macrophages and monocytes (Ransohoff and Cardona, 2010). Microglia express CD11b/c, D45^low^, and the chemokine fractalkine receptor (CX3CR1) (Dudvarski Stankovic et al., 2016). These cells survey the CNS in order to detect homeostasis alteration and they respond accordingly, combining a defensive service with neuroprotective functions (Verkhratsky and Butt, 2013).

In addition to the immune functions, microglia have an essential role in the physiology and survival of neurons. Fractalkine, involved in indirect neuroprotection, is released by neurons and the receptor is expressed by microglia, their interactions constituting a neuron-microglial signaling system (Ransohoff and El Khoury, 2015). The fractalkine expressed by neurons can induce adenosine release from microglia. This adenosine (via adenosine A1 receptor) can activate survival pathways in neurons sensitive to excitotoxicity challenge (Lauro et al., 2008, 2010). The signaling mediated by CX3CR1 could regulate microglial behavior in the neurodegenerative diseases.

The presence of protein aggregates in the CNS is a common feature of most neurodegenerative disorders. These aggregates are identified by the Toll-like receptors (TLRs) which are danger-signal sensors. Microglial cells express these receptors (TLR1-TLR9) and their co-receptors, which promote microglial activation (Gonzalez et al., 2014). Concretely, TLR4 and TLR2 are associated with both neuro-inflammation and clearance of protein aggregates in neurodegenerative disorders (Jack et al., 2005).

Microglia constitute the first line of immune defense in CNS. After injury these cells become activated and in this state change their morphology, proliferate, migrate to the damage sites, modify the expression of enzymes and receptors, and release a variety of inflammatory factors, such as NO, tumor necrosis factor (TNF-α), interleukin (IL-6) among others (Magni et al., 2012). The morphology of activated microglia includes a retraction of processes, enlargement of the soma, and increased expression of myeloid cell markers (Ransohoff and Cardona, 2010). In their state of high activation, microglial cells acquire an amoeboid morphology and act like macrophages, engulfing debris (Brown and Neher, 2014). Excessive microglia activation might prompt the release of cytotoxic factors, causing neuronal damage, which could accelerate the progression of some CNS diseases.

Microglial cells may undergo two different kinds of activation in response to infections or injuries. The first is a neurotoxic phenotype called M1-like. This phenotype generates a massive inflammatory response releasing interleukin-1β (IL-1β), IL-12, TNF-α and inducible nitric oxide synthase (iNOS). M1 microglial cells present amoeboid morphology as well as high phagocytic capacity and motility (Varnum and Ikezu, 2012; Gonzalez et al., 2014; Jones and Bouvier, 2014). However, in certain circumstances, the neuroinflammation can help stimulate myelin repair or remove toxic aggregated proteins and cell debris from CNS (Ding et al., 2004; Simard et al., 2006; Glezer et al., 2007). After this acute M1 activation, microglial cells can suffer an uncontrolled activation leading to a state of chronic inflammation. In this state, microglia release neurotoxic inflammatory factors (TNF-α, IL-1α, IL-1β, IL-6, NO, hydrogen peroxide, superoxide anion, chemokines, and glutamate), which lead to neuronal death (Block et al., 2007; Lull and Block, 2010; Burguillos et al., 2011; Kettenmann et al., 2011; Gordon et al., 2012).

The second microglial phenotype, M2-like, secrete anti-inflammatory mediators and neurotrophic factors, thus inducing a supportive microenvironment for neurons (Kettenmann et al., 2011). The M2 microglial cells are characterized by thin cellular bodies and ramified processes (Menzies et al., 2010; Komori et al., 2011; Varnum and Ikezu, 2012; Jones and Bouvier, 2014; Zhou et al., 2014). These cells can release anti-inflammatory cytokines including IL-4, IL-13, IL-10, TGF-β and neurotrophic factors, such as insulin-like growth factor 1 (IGF-1) to assist inflammation resolution and promote neuron survival (Suh et al., 2013; Tang and Le, 2016). M2 microglia are the major effector cells with the potential to dampen pro-inflammatory immune responses and promote the expression of repair genes (Tang and Le, 2016). The change of microglia between M1 and M2 phenotypes is a dynamic process and microglial activation can switch from M2 to M1 phenotype during the course of disease (Cherry et al., 2014).

Recently, it has been reported that microglia release extracellular microvesicles (Evs) by exocytosis. These microvesicles are involved in all immune activities and can be protective or detrimental, affecting some pathologies of the CNS. EVs have a heterogeneous molecular composition, including receptors, integrins and cytokines, bioactive lipids, miRNA, mRNA, DNA, and organelles, being similar to their parental cells. They can be detected in the plasma and other biological fluids such as the cerebral spinal fluid (CSF). The microglial EVs representing a “liquid biopsy” of their parental cells, and could provide information on the functional phenotype (protective or damaging) of microglial cells over the course of neurodegeneration (Nigro et al., 2016).

Microglia and astrocytes are the main innate immune effector cells in the CNS (da Fonseca et al., 2014). Under pathological conditions, astrocytes and microglia can collaborate to induce an inflammatory response. After injury, astrocytes produce cytokines and chemokines (CCL2, CXCL1, CXCL10, GM-CSF, and IL-6), which activate microglia and recruit peripheral immune cells to the CNS. By contrast, a recent report has described an astrocyte subtype A1 that is abundant in AD and PD and other human neurodegenerative diseases. Activated microglia can induce A1 astrocytes by secreting IL-1α, TNF, and C1q, and this type of astrocyte contribute to the death of neurons in the neurodegenerative disorders and could be analog to the M1-like phenotype microglia (Liddelow et al., 2017).

Microglia, together with endothelial cells, pericytes, and astrocytes, form the functional blood-brain barrier (BBB) that selectively separates the brain parenchyma from blood circulation. In this perivascular location, the microglia survey the influx of blood-borne components entering the CNS. The activated microglia can induce the dysfunction of the BBB, being correlated with the disruption of the BBB in the neurodegenerative diseases (Dudvarski Stankovic et al., 2016). During inflammatory conditions, innate immune cells (DCs, neutrophils, monocytes, and natural killer cells) and adaptive immune cells (activated B cells together with CD4+ and CD8+ T cells) are recruited by chemoattractants to cross the BBB from the periphery. The presence of this cellular infiltrate in the CNS can directly or indirectly provoke neuroinflammation by producing pro-inflammatory cytokines/chemokines. All this could generate oxidative stress, which leads to neuronal death. In addition, activated microglia are capable of upregulating CD11c, MHC I, and MHC II to act as antigen-presenting cells, which activate T cells. This activation would in turn damage the nervous system (Xu et al., 2016).

As mentioned above, during the inflammatory process, there is a are released of cytokines. Cytokines bind to receptors in the microglia and activate the JAK/STAT signaling pathway (Yan et al., 2016). This pathway plays a critical role in the initiation and regulation of innate immune responses and adaptive immunity (Yan et al., 2016). Although the same JAK/STAT components are used, the gene expression in response to a specific cytokine, depending on the cell type (van Boxel-Dezaire et al., 2006). This pathway constitutes a pattern-recognition system by which microglial cells respond to foreign antigens and inflammation in the CNS (Hanisch and Kettenmann, 2007).

In microglial cells, other receptors called the “triggering-receptors-expressed-on-myeloid-cells” (TREM) are thought to play a central role in the immune-system regulation and inflammation. The signaling pathway TREM2 regulates apoptosis, the immune response, and phagocytic activity. Brain homeostasis without inflammation depends on eliminating extracellular aggregates and apoptotic debris, this being mediated by the TREM2/DAP12 receptor complex (Han et al., 2017). The signaling pathway TREM2 regulates the apoptosis, the immune response, and the phagocytic activity. This pathway, induced in the microglial cells by anti-inflammatory cytokines, is modulated by CD33 and is down-regulated by agonists of TLR2, 4, and 9, as well as by inflammatory stimuli such as lipopolysaccharides and RNA interference. An overexpression of TREM2 promotes phagocytosis and reduces the pro-inflammatory response (Han et al., 2017). This receptor is a critical regulator of microglia and macrophage phenotype and is involved in neurodegenerative diseases (Andreasson et al., 2016).

After damage, microglia transform into active phagocytes. These cells migrate to the damaged area and adopt an amoeboid morphology, releasing both pro- and anti-inflammatory molecules. They also have the capacity to remove apoptotic cells and debris. As mentioned above, for phagocytosis to occur the expression of specific receptors on the microglial surface is necessary. The principal receptors are the TLRs, which have high affinity for pathogens, and TREM2, which recognizes apoptotic cellular substances (Hsieh et al., 2009). In addition, other receptors also participate in cell-debris clearance (Fc receptors, complement receptors, scavenger receptors (SR), pyrimidinergic receptors P2Y, G-protein coupled,6 (P2RY6), macrophage antigen complex 2 (MAC-2), mannose receptor, and low-density lipoprotein receptor-related protein (LRP) (Fu et al., 2014).

Although microglia are the main agents responsible for phagocytosis of cell debris in the CNS, the complement system can play a primordial role in removing damaged and apoptotic cells (Fakhoury, 2016). Microglial cells can activate the complement by local secretion of the complement component from both the classical and the alternative pathway and also express C3 and C5 (Luo et al., 2011). The complement also participates in the physiological process, termed synaptic pruning. The synapses and axons have to be labeled by complement components C1q and C3 before being phagocytosed, which prompts their selective recognition by microglial cells (Paolicelli et al., 2011; Linnartz et al., 2012; Schafer et al., 2012).

Given the central role of microglial cells in neurodegeneration, the evaluation of activated microglia *in vivo* is an important approach. Positron emission tomography (PET) is the most widely used *in vivo* method for detecting microglial activation (Owen and Matthews, 2011; Mirzaei et al., 2016). It has been found that activated microglia and astrocytes overexpress mitochondrial translocator protein (TSPO) within or surrounding senile plaques. Thus, it has been proposed that neuroimaging of TSPO using PET is a good marker of neuroinflammation (Cosenza-Nashat et al., 2009; Pasqualetti et al., 2015).

## AD and microglia

The primary pathogenic process in AD is the accumulation of Aβ protein. This protein aggregates into extracellular amyloid plaques, which are the hallmark of this pathology (Southam et al., 2016). The amyloid hypothesis for AD is based on a linear, quantitative, centered neuron model. This model postulates that the initial deposition of Aβ triggers mechanisms that progressively lead to Tau pathology, synaptic dysfunction, inflammation, neuronal loss and finally to dementia (De Strooper and Karran, 2016). Recently, evidence has been reported that Aβ protein acts by increasing tau pathology through the formation of tau species capable of producing new aggregates (Bennett et al., 2017).

After acute inflammatory damage, the brain glial cells respond to repair the tissue. If the stimulus persists, it produces an inflammatory chronic state that leads to neuronal dysfunction, injury, and loss (Streit et al., 2004; Calsolaro and Edison, 2016). As mentioned before, inflammation is one of the possible causes in the development of AD (Wyss-Coray, 2006). The increase in Aβ deposition induces the activation of astrocytes as well as microglia (Cagnin et al., 2001). These activated cells can release both pro- and anti-inflammatory mediators, leading to a state of chronic inflammation in the tissue. This inflammation not only occurs in response to Aβ deposition, but is also capable of generating, via feedback mechanisms, more Aβ while weakening the mechanisms responsible for its elimination (Parpura et al., 2012).

The Soluble Aβ oligomers and Aβ fibrils can react to various receptors expressed by microglia, including CD14, CD36, CD47, α6β1 integrin, class A scavenger receptor, receptor for advanced glycosylation end products (RAGE) and TLRs (Stewart et al., 2010). The RAGE is an important cell-surface receptor for Aβ in the endothelial cells, neurons, and microglia, and increased expression in these cell types has been demonstrated in AD (Yan et al., 1996). The interaction of Aβ with RAGE causes oxidative stress in neurons, enhances inflammatory responses in microglia, and is involved in reversed transport of Aβ across the BBB in endothelial cells (Deane et al., 2012). The binding of Aβ to CD36 (cell surface microglial co-receptor) promotes the TLR4 and/or TLR6 phosphorylation and activation, resulting in the production of inflammatory cytokines and chemokines (Stewart et al., 2010).

Recently, an alternative pathway has been described for intracellular signaling produced by the binding of Aβ to microglial cells, activating NLRP3 inflammasome (Heneka et al., 2013; Sheedy et al., 2013). NLRP3 inflammasome is an intracellular protein complex. Their assembly and activation regulates activation of caspase-1, which catalyzes the cleavage and activation of proinflammatory cytokines of the IL-1β family, promoting the secretion of these, now biologically active, cytokines. These cytokines could induce neuronal degeneration (Gold and El Khoury, 2015). In addition, NLRP3 inflammasome activation reduces phagocytosis of Aβ by microglial cells, thus increasing the Aβ depositions and contributing to the pathogenesis of AD (Garlanda et al., 2013; Gold and El Khoury, 2015).

Microglial cells are important for the normal functioning of neurons in the CNS. They provide trophic support to neurons and regulate synapses. The altered microglial behavior could induce neuronal degeneration in AD (Southam et al., 2016). During development, microglia are involved in synapse elimination and these mechanisms may be aberrantly reactivated in the aged brain, contributing to the synapse loss in AD. The synapse loss in the hippocampus and association cortices is an early hallmark of AD and strongly correlates with cognitive impairment (Hong et al., 2016). In the healthy development of the brain, the proteins of complement participate in synapse pruning. Synapses to be cleared express C3 and binding to CR3 on microglia, resulting in microglial phagocytosis of the synapse (Schafer et al., 2012; Southam et al., 2016). In the healthy adult brain, these complement components are downregulated. However, in aging brains, C1q and C3 are highly upregulated and are deposited on synapses, particularly in the hippocampus, the most vulnerable region in the synapse loss in AD (Bialas and Stevens, 2013). These findings highlight the importance of complement regulation for normal synaptic maintenance (Southam et al., 2016). In addition, it has been shown that Aβ can bind and regulate the expression and localization of complement proteins in the AD brain. An upregulation has been observed in the complement proteins (C1q, C3, and C4) localized in senile plaques also known as neuritic plaques (Hong et al., 2016).

Microglial cells can use additional mechanisms for synapsis regulation. The release of brain-derived neurotrophic factor (BDNF) by microglial cells induces synaptic pruning. However, the depletion of this factor in the microglia results in learning and memory impairment (Parkhurst et al., 2013). The activation of the fractalkine receptor (CX3CR1) in microglial cells, increases synaptic strength. However, deficiency in this receptor results in a reduced hippocampal synaptic plasticity (Rogers et al., 2011; Clark et al., 2015).

It has been reported that in the later stages of AD, there is destruction of axons, dendrites, and synapses, in which microglia has a relevant role (Parpura et al., 2012). In AD brains reactive microglia has been found colocalized with amyloid plaques. In addition, the reactive astrocytes accumulate around senile plaques next to the activated microglia (Heneka et al., 2015). In brain, astrocytes as well as microglia are capable of capturing Aβ for degradation (Pihlaja et al., 2011). In AD patients, astrocytes in the entorhinal cortex accumulate Aβ, this accumulation being positively correlated with the extent of AD (Nagele et al., 2003). Moreover, astrocytes can also induce microglia to perform Aβ phagocytosis by regulating the release of the apo E and the ATP-binding cassette (ABCA) protein. Studies *in vitro* have demonstrated that microglial phagocytosis of Aβ is more effective in the presence of supernatants derived from astrocytes (Terwel et al., 2011). In AD, mutations in ABCA7 can cause a loss of receptor activity, resulting in reduced microglia phagocytic function (Southam et al., 2016).

Microglial senescence can enhance the sensitivity of microglia to inflammatory stimuli; this phenomenon is called “priming” (Heneka et al., 2015). In addition, aged microglia show reduced phagocytic capacity. This process could be due partly to a reduction in the ability of microglia to recognize phagocytic targets (Udeochu et al., 2016). Both inflammation and reduced microglial phagocytic capacity in AD can contribute to the decline in synaptic plasticity observed in this pathology (Ritzel et al., 2015; Udeochu et al., 2016).

In AD, protein aggregation is caused by declining of protein homeostasis (proteostasis) (Mosher and Wyss-Coray, 2014). As mentioned above, Aβ deposits can attract and activate microglia. Presumably, microglial proliferation around plaques could serve as a line of defense to limit the deposition of amyloid. Nonetheless, it seems that microglial cells clustered around Aβ− deposits have become incapable of removing the amyloid (Calsolaro and Edison, 2016). The sustained exposure to cytokines, chemokines and Aβ, could be responsible for the functional impairment of microglial cells located around Aβ− deposits (Heneka et al., 2015). In addition, microglial-specific genetic alterations may be related to this microglial dysfunction. The expression of beclin 1, a protein associated with autophagy pathway, is reduced in the brain of patients with AD, leading to disruption in phagocytosis and retromer-mediated recycling of the phagocytic receptors CD36 and TREM2 in microglia (Mosher and Wyss-Coray, 2014).

Similarly, mutations in TREM2 can trigger the loss of phagocytic capacity in microglial cells. TREM2 inhibits pro-inflammatory cytokine production, facilitates phagocytosis and promotes cell survival. Thus, TREM2 dysfunction could induce the loss of the homeostasis in the tissue (Painter et al., 2015). Missense mutations in TREM2 lead to a significant risk of developing AD (Jonsson et al., 2013; Meyer-Luehmann and Prinz, 2015).

As mentioned above, in AD the Aβ peptide that is aggregated extracellularly in the neuritic plaques produces an inflammatory environment and a chronic activation of microglial and astroglial cells (D'Andrea, 2005). Activated microglia can shed MVs in response to several signals, including cytokines. These MVs contain bioactive molecules (i.e., IL-1β, proteases, and MHC-II) which modulate the activity of neuronal and non-neuronal cells (Antonucci et al., 2012). In AD patients, the production of MVs is very high, reflecting microgliosis. These extracellular vesicles can be isolated form cerebrospinal fluid (Guerriero et al., 2016).

In patients with AD, an upregulation of iNOS has been found. In the course of AD, cytokines stimulate iNOS in microglia and astrocytes, generating high NO levels (Vodovotz et al., 1996). NO can interact with signaling cascades and regulate gene transcription, impair mitochondrial respiration or directly induce neuron death by apoptosis or necrosis (Parpura et al., 2012). In addition, the NO can promote the nitration of Aβ, increasing their propensity to aggregate (Kummer et al., 2011; Heneka et al., 2015).

In late-onset Alzheimer's disease (LOAD), accumulating Aβ and NO harm the cells of the cerebral vessel, causing the onset of cerebral amyloid angiopathy (Nelson et al., 2016). The neurovascular unit, constituted by cerebral blood vessels, perivascular glia and neurons, are associated with distinct inflammatory, functional, and morphological alterations in AD (Heneka et al., 2015). In LOAD, damaged blood vessels can hinder neurogenesis from neural stem cells in the subventricular zone and hippocampus, preventing the processing and storage of new memories (Licht and Keshet, 2015; Chiarini et al., 2016).

Recently, it has been suggested that the involvement of glial cells in AD is related with the transient receptor potential melastatin member 2 (TRPM2). This receptor, besides regulating synaptic plasticity and glial cell activation, also modulates oxidative stress and inflammation (Yuruker et al., 2015). TRPM2 channel can be activated by Aβ. The activation of these channels in microglia and astrocytes leads to Ca^2+^ overload and subsequent inflammation and oxidative stress. All of this causes mitochondrial dysfunction, [Ca^2+^]_I_ increase, Aβ accumulation, glutamate-receptor dysfunction, and finally plasticity alterations and dementia (Yuruker et al., 2015; Wang et al., 2016).

## Parkinson and microglia

PD is characterized by α-synuclein (α-syn) accumulation, dopaminergic neuron loss and inflammation (Beach et al., 2014; Wang et al., 2015). The pathological hallmark of this disorder is the presence of Lewy bodies. The Braak hypothesis has suggested that PD begins in the olfactory bulb or the gastrointestinal tract. These areas are constantly exposed to the environment, and in them, the Lewy bodies accumulate (Kannarkat et al., 2013). Lewy bodies are constituted mainly by misfolded α-syn and other intraneuronal protein aggregates such as tau and ubiquitin proteins (Campello et al., 2013; George and Brundin, 2015). The nitration, phosphorylation, and ubiquitination of α-syn can promote their pathological accumulation, inducing neurodegeneration (Giasson et al., 2000; Tofaris et al., 2003; Anderson et al., 2006). In addition, missense mutations in α-syn can produce the protein aggregation in familial PD (Conway et al., 1998).

Reportedly, α-syn can induce microglial activation, which in turn can promote α-syn phagocytosis (Cao et al., 2012) and neuroinflammation. The neuroinflammation leads to the loss of dopaminergic neurons and drives the chronic progression of neurodegeneration in PD (Schapansky et al., 2015). Accumulations of activated microglia have been found around dopaminergic neurons in postmortem human brains (Hamza et al., 2010). Microglial cells can be activated by α-syn, via TLRs, initiating an immune response (Fellner et al., 2013). Specifically, the stimulation of TRL2 and TRL4 in the microglia induces signaling cascades involved in the inflammatory response. It has been shown in PD patients that TLR2 colocalized with CD68+ amoeboid microglia indicates microglial activation at the sites of neuronal loss (Doorn et al., 2014). Also, TRL4 can induce microglial phagocytosis of α-syn. Deficiencies in this receptor can prompt poor α-syn clearance and neurodegeneration (Fellner et al., 2013).

The clearance of α-syn also can be promoted by the leucine-rich repeat kinase 2 (LRRK2) gene. This gene has been proposed as a regulator of the microglial response (Schapansky et al., 2015). LRRK2 is the most commonly mutated gene in both idiopathic and familial PD. Pathogenic mutations in LRRK2 influence the ability of microglia to internalize and degrade α-syn, exacerbating α-syn-induced microglial pathology, and neuroinflammation (Schapansky et al., 2015). In addition, other genes whose mutations are responsible for rare familial forms of PD have been identified, including, SNCA, PARKIN, DJ-1, and PINK1 (Chao et al., 2014).

Persistent microglial activation is known to exert harmful effects that result in dopaminergic neuron death. One of the most important signaling pathways associated with the microglial activation in PD involves nuclear factor-kappa B (NF-kB) (Zhang et al., 2017). The activation of this factor could increase the release of proinflammatory cytokines such TNF-α and interleukin 1β by microglial cells (Mogi et al., 1996; McLaughlin et al., 2006). In addition, proinflammatory mediators such as TNF-α, IL-1β, and IFN-γ have been found at higher levels in the midbrain of PD patients (Wang et al., 2015). Immunomodulators, including the CX3CL1, CD200, CD22, CD47, CD95, and neural cell adhesion molecule, sustain the rest state of microglia under normal conditions (Chang et al., 2000; Sheridan and Murphy, 2013). In rat PD models, both deficiency CX3CL1 or CX3CR1 as well as the dysfunction of CD200-CD200R signaling have been shown to increase microglial activation and the degeneration of DA neurons (Wang et al., 2011; Zhang et al., 2011).

The cytokines released by activated microglia can attract peripheral immune cells (e.g., CD4 T-cell) to the brain. *In vivo* and *in vitro* studies have demonstrated that overexpression of α-syn can induce the MHC-II expression by microglia. The MHC-II expression in microglia cells can play an important role in the immune responses (innate and adaptive) in PD (Michelucci et al., 2009; Harms et al., 2013; Gonzalez et al., 2015).

In addition, dopaminergic neurons seem to be especially sensitive to several factors that can induce cell damage and eventually cell death. It has been suggested that mitochondrial malfunction leads to reduced energy metabolism and induces neuroinflammation via NO and ROS production, which ultimately entails neurodegeneration (Vivekanantham et al., 2015). The production of NO and superoxide exerted by activated microglia in PD can cause the degeneration of dopaminergic neurons (Appel et al., 2010). The high cytosolic concentrations of free DA can produce oxidative stress and can interact with α-syn, promoting the neurodegenerative process (Mosharov et al., 2006). In addition, the neuromelanin (dark, complex endogenous polymer derived from DA) can activate microglial cells, inducing neuroinflammation and neurodegeneration of dopaminergic neurons in PD (Zecca et al., 2008; Herrera et al., 2015).

Neuroinflammation is produced by the set of integrated responses of all the CNS immune cells including microglia, astrocytes and infiltrating T-lymphocytes (Le et al., 2016). Gliosis in the PD is an atypical activation where astrogliosis is largely absent while the microglia is highly activated by the disease. The low astroglial response may be caused by degeneration due to an increase of α-syn in the astrocyte (Stefanova et al., 2001; Orr et al., 2002; Sofroniew and Vinters, 2010). Astrocytes are responsible for secreting glutathione and transporting to neurons in response to neural excitatory stimuli. A lower level of glutathione has been detected in the CNS of PD patients, and thus the antioxidant capacity in the tissue could be impaired, probably secondary to the astroglial defect (Olanow and Tatton, 1999).

Recently, the kinurenic pathway (KP) has been implicated in the inflammatory and neurotoxic processes in PD. Astrocytes produce a neuroactive component of KP, kynurenic acid, considered to be neuroprotective. By contrast, quinolinic acid, released by microglia, can activate the NMDA receptor-signaling pathway, leading to excitotoxicity and increasing the inflammatory response. Based on this, KP may represent an important target to prevent the progression of the underlying neurodegeneration observed in PD (Lim C. K. et al., 2017).

Nowdays, it has been reported that prothrombin kringle-2 (pKr-2), which is a domain of prothrombin (which is produced by active thrombin), could be involved in PD. Also, pKr-2 induced DA neuronal death in an experimental PD model (Kim et al., 2010). In addition, in PD patient's pKr-2 expression is significantly increased and co-localized in activated microglial in the substantia nigra, leading to disruption of the nigrostriatal DA projection. This disruption could be mediated through the neurotoxic inflammatory events brought about by the pKr-2 upregulation, wich trigger microglial activation via TLR4. On the basic of these results, limiting pKr-2-induced microglial activation may be an effective therapeutic strategy for protecting DA neurons (Leem et al., 2016).

## Neurodegenerative diseases and the eye

### Alzheimer's disease

**Figure 1:**
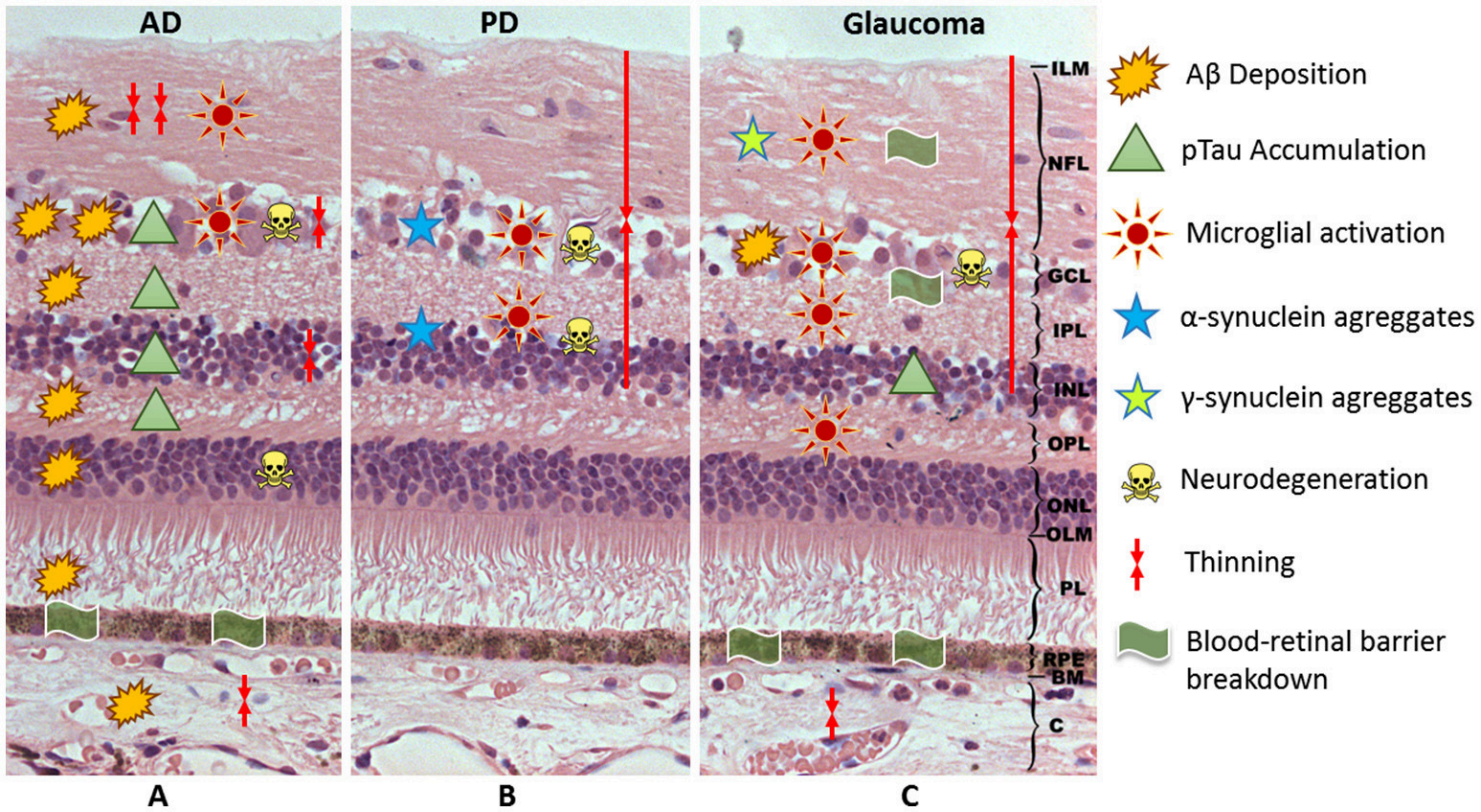
Schematic representation of the hypothetical events associated with the neuroinflammation in AD **(A)**, PD **(B)**, and glaucoma **(C)**. AD, Alzheimer's Disease; PD, Parkinson's Disease; ILM, inner limitant membrane; NFL, nerve fiber layer; GCL, ganglion cell layer; IPL, inner plexiform layer; INL, inner nuclear layer; OPL, outer plexiform layer; ONL, outer nuclear layer; OLM, outer limitant membrane; PL, photoreceptor layer; RPE, retinal pigment epithelium; BM, Bruch membrane; C, choroid; Aβ, beta-amyloid; pTau, phosphorylated tau.

Classically, the damage in AD was thought to be restricted mainly to the brain. However, in the last few decades it has been demonstrated that patients with AD often develop visual anomalies, which are correlated with abnormalities in the eye. Among them, there is a reduction in the number of optic nerve head axons and a decrease in the thickness of the peripapillary and macular retinal nerve fiber layer (RNFL) (Tsai et al., 1991; Hedges et al., 1996; Danesh-Meyer et al., 2006; Iseri et al., 2006; Paquet et al., 2007; Garcia-Martin et al., 2014; Salobrar-Garcia et al., 2015; Salobrar-García et al., 2016c) (Table 1). One of the earliest symptoms of AD could be the thinning of the RGC layer and visual spatial impairment (Kesler et al., 2011). Postmortem studies in AD retinas, have demonstrated that, in addition to RGC loss, melanopsin retinal ganglion cells (mRGC) are lost. There is evidence that mRGCs may be affected primarily by Aβ pathology in AD (La Morgia et al., 2011). This mRGC deficiency could be correlated with a circadian dysfunction (La Morgia et al., 2011) in which AD patients tend to be more active during the night in comparison with the day (Hatfield et al., 2004; Hooghiemstra et al., 2015). In addition, in the retina of AD patients as well as AD human postmortem specimens the presence of Aβ plaques has been demonstrated. Aβ deposition was observed from the outer nuclear layer (ONL) to nerve fiber layer (NFL), being more abundant in the superior region of the retina where greater neuronal degeneration has been detected (Hardy and Selkoe, 2002; Selkoe, 2004, 2008; Alexandrov et al., 2011; Ratnayaka et al., 2015; Hart et al., 2016; Table 1). In AD patients, the alloform Aβ42 is increased (Alexandrov et al., 2011). This alloform presents higher cellular toxicity, more aggregation capacity, and a more direct relation with AD pathology (Qiu et al., 2015). Aβ42 peptide accumulation in the retina may contribute to retinal degeneration and visual impairment in AD (Hardy and Selkoe, 2002; Selkoe, 2004, 2008; Alexandrov et al., 2011; Ratnayaka et al., 2015; Hart et al., 2016; Figure 1A, Table 1). However, recently Williams et al. in AD patients found no evidence of deposits or accumulations of Tau, Aβ, TDP-43, ubiquitin or α-syn in any part of the eyeball (Williams E. A. et al., 2017).

**Table 1:** Retinal changes associated with AD, PD, and glaucoma.

		**AD**	**PD**	**Glaucoma**
		**References**		
Retinal thickness decrease		Tsai et al., 1991; Hedges et al., 1996; Danesh-Meyer et al., 2006; Iseri et al., 2006; Paquet et al., 2007; Kesler et al., 2011; Garcia-Martin et al., 2014; Maldonado et al., 2015; Salobrar-Garcia et al., 2015, 2016a,b; Salobrar-García et al., 2016c	Inzelberg et al., 2004; Yu et al., 2014; Stemplewitz et al., 2015; Boeke et al., 2016; Satue et al., 2017	Leung, 2016; Fallon et al., 2017
Inner retinal involvement		La Morgia et al., 2011	Bodis-Wollner, 1990; Tatton et al., 1990; Surguchov et al., 2001; Cuenca et al., 2005; Hajee et al., 2009; Bodis-Wollner et al., 2014	Rojas et al., 2014
Outer retinal involvement		Hardy and Selkoe, 2002; Selkoe, 2004, 2008; Ratnayaka et al., 2015; Hart et al., 2016	Maurage et al., 2003; Esteve-Rudd et al., 2011	
Protein deposits in retina	Aβ	Hsiao et al., 1996; Holcomb et al., 1998; Takeuchi et al., 2000; Lukiw et al., 2001; Hardy and Selkoe, 2002; Oddo et al., 2003; Selkoe, 2004, 2008; Kumar-Singh et al., 2005; Oakley et al., 2006; Philipson et al., 2010; Alexandrov et al., 2011; Koronyo-Hamaoui et al., 2011; Kayabasi et al., 2014; Qiu et al., 2015; Ratnayaka et al., 2015; Hart et al., 2016		McKinnon et al., 2002; McKinnon, 2003; Goldblum et al., 2007
	pTau	Liu et al., 2009; Gasparini et al., 2011; Lim J. K. et al., 2016		Gupta et al., 2008; Ning et al., 2008; Bolos et al., 2017
	α-syn		Surguchov et al., 2001; Maurage et al., 2003; Bodis-Wollner et al., 2014	
	γ-syn			Surgucheva et al., 2002
Microglial activation		Ning et al., 2008; Perez et al., 2009; Parnell et al., 2012	Chen et al., 2003; Nagel et al., 2009; Cho et al., 2012	Kreutzberg, 1995; Neufeld et al., 1997; Giulian and Ingeman, 1988; Wax et al., 1998; Neufeld, 1999; Shareef et al., 1999; Tezel et al., 2001; Yang et al., 2001b; Yuan and Neufeld, 2001; Naskar et al., 2002; Steele et al., 2005; Taylor et al., 2005; Nakazawa et al., 2006; Stasi et al., 2006; Vidal et al., 2006; Farina et al., 2007; Inman and Horner, 2007; Johnson et al., 2007; Hammam et al., 2008; Tezel, 2009; Ebneter et al., 2010; Graeber and Streit, 2010; Luo et al., 2010; Bosco et al., 2011, 2012; Kettenmann et al., 2011; London et al., 2011; Bosco et al., 2012; Gallego et al., 2012; Varnum and Ikezu, 2012; de Hoz et al., 2013; Gramlich et al., 2013; Pinazo-Duran et al., 2013; Astafurov et al., 2014; Cherry et al., 2014; Gonzalez et al., 2014; Jones and Bouvier, 2014; Lee et al., 2014; Rojas et al., 2014; Karlstetter et al., 2015; Madeira et al., 2015; Ransohoff and El Khoury, 2015; Chidlow et al., 2016; Bolos et al., 2017; Williams E. A. et al., 2017
Neurodegeneration		Hatfield et al., 2004; Schlamp et al., 2006; Leroy et al., 2007; Liu et al., 2009; La Morgia et al., 2011; Veerhuis, 2011; Parnell et al., 2012; Tsai et al., 2014; Hooghiemstra et al., 2015	Bodis-Wollner, 1990; Tatton et al., 1990; Cuenca et al., 2005	Neufeld, 1999; Yuan and Neufeld, 2001; Steele et al., 2005; Garden and Möller, 2006; Quigley and Broman, 2006; Stasi et al., 2006; Koizumi et al., 2007; Langmann, 2007; Ohsawa et al., 2007; Wu et al., 2007; Tezel, 2009; Karlstetter et al., 2010, 2015; Taylor et al., 2011; Rojas et al., 2014; Wang et al., 2014; Bosco et al., 2016; Chidlow et al., 2016
Blood-retinal barrier breakdown				Farina et al., 2007; Tezel, 2009; London et al., 2011; Howell et al., 2012; Gonzalez et al., 2014; Karlstetter et al., 2015; Breen et al., 2016
Visual impairment		Krasodomska et al., 2010; Tsai et al., 2014	Djamgoz et al., 1997	

*AD, Alzheimer's Disease; PD, Parkinson's Disease; Aβ, beta-amyloid; pTau, hyperphosphorylated tau protein; α-syn, α-synuclein; γ-syn, γ–synuclein*.

Aβ plaques have also been detected in the retina of transgenic mouse models of AD (APP_swe_ / PS1_ΔE9_, Tg2576AD, 3xTg-AD, PSAPP, 5xFAD; Hsiao et al., 1996; Holcomb et al., 1998; Takeuchi et al., 2000; Lukiw et al., 2001; Oddo et al., 2003; Kumar-Singh et al., 2005; Oakley et al., 2006; Philipson et al., 2010; Koronyo-Hamaoui et al., 2011). Overall, in these mice, Aβ plaques were found principally in the NFL, ganglion cell layer (GCL), inner plexiform layer (IPL), inner nuclear layer (INL) and outer plexiform layer (OPL) (Table 1). In APP_swe_ / PS1_ΔE9_ transgenic mice, Aβ plaques appeared in the retina of young transgenic AD mice at presymptomatic stages, as early as 2.5 months, preceding their detection in the brain. This situation points out a correlation between retinal and brain pathology in AD. The detection of retinal Aβ might potentially provide an alternative noninvasive approach to assess the progression of AD. In relation to this last point, it has been demonstrated that the systemic administration of curcumin to AD mice resulted in specific *in vivo* labeling of retinal Aβ plaques. This finding provides the basis for the development of a high-resolution noninvasive optical-imaging technique for detecting Aβ plaques in the retina, allowing the early diagnosis and follow up of AD (Koronyo-Hamaoui et al., 2011; Kayabasi et al., 2014). In addition, curcumin has been revealed to be a novel agent for treating AD through different neuroprotective mechanisms, such as inhibition of Aβ aggregation and decrease in neuroinflammation (Maiti and Dunbar, 2016; Lakey-Beitia et al., 2017).

In addition to Aβ plaques, pTau was observed from OPL to GCL in the retina of AD patients. Also, pTau has been found from the ONL to GCL in the transgenic mouse (Liu et al., 2009). It has been postulated that pTau could be potentially a marker for the AD disease (Lim J. K. et al., 2016). In a model of transgenic mice P301S tau, early accumulation of pTau and βIII-tubulin in the NFL of the retina was demonstrated. This accumulation was accompanied by somatodendritic redistribution of pTau and the subsequent development of tau inclusions in a group of RGCs. In the optic nerve of this transgenic model, at 5 months of age, damaged axons were detected presenting phospho-tau, neurofilaments, amyloid precursor protein and ubiquitin accumulations, as well as disordered filaments and degenerating mitochondria and organelles (Gasparini et al., 2011). These observations suggest that tau may alter axonal transport. This alteration is an early event in tau-induced neuronal dysfunction and corroborates previous findings in mouse models of tautopathy and glaucoma, showing that axonal degeneration precedes neuronal loss (Schlamp et al., 2006; Leroy et al., 2007; Figure 1A, Table 1).

A significant upregulation of inflammation (evidenced by astroglial and microglial activation) has been found in the retinas of AD mouse models in relation to Aβ plaques (Parnell et al., 2012). Ning et al. (2008) observed an age-dependent increment in Aβ in the retina of the double transgenic mice model APP_swe_ / PS1_ΔE9_. This increment was accompanied by increases in the inflammatory cytokine MCP-1, the microglial marker F4/80, and the TUNNEL-positive cells in the RGC layer. Thus, the authors suggested that Aβ played a major role in the inflammation and neurodegeneration in AD. In the same transgenic model, Perez et al. (2009) observed significantly greater microglial activity. Microglial activation could occur early in the retina and could be involved in the elimination or turnover of Aβ deposition. In addition, activated microglia could trigger a neuroinflammatory response, which may contribute to a disorganization of the retina, as demonstrated by electroretinogram functional alterations (Krasodomska et al., 2010; Table 1). This neuroinflammatory response associated with Aβ plaques and pTau, has also been observed in Tg2576AD mice. In these animals, there was a significant increase in Iba1 cells (a microglial marker) and an increase in the glial fibrillary acidic protein (GFAP) immunoreactivity (a marker of astrocytes) (Figure 1A). The vaccination with Aβ oligomer antigen reduced Aβ retinal deposits in these transgenic mice. However, the microvascular Aβ deposition as well as the microglial infiltration and astrogliosis were increased and were associated with the disruption of retinal architecture (Liu et al., 2009). Other studies support the involvement of the neuroinflammation in the AD progression. These studies analyzed the role of the complement in this disease (Parnell et al., 2012). Deficits have been found in the expression of the innate immune-repressor complement factor H (CFH) associated with significant increases of Aβ42 peptides in brains and retinas of transgenic models of AD (Veerhuis, 2011). CFH functions as a cofactor in the inactivation of C3b in the alternative complement pathway, and thus low CFH levels result in complement activation, triggering inflammation in the retina and brain (Alexandrov et al., 2011). In the transgenic rat model (TgF344-AD) also has been observed, along with Aβ deposition, microglial recruitment, and complement activation in association with a decline in visual function (Tsai et al., 2014; Table 1).

### Parkinson's disease

As mentioned above, PD is a motor disorder associated with degeneration of dopaminergic neurons in the substantia nigra (Inzelberg et al., 2004). In this disease, high levels of α-syn are found in midbrain dopaminergic neurons (Neystat et al., 1999; Solano et al., 2000; Braak et al., 2003; Kingsbury et al., 2004; Alafuzoff and Parkkinen, 2014). Moreover, abnormalities in visual function have been reported (Bodis-Wollner, 1990; Nowacka et al., 2014) in PD patients and correlated with changes in retinal tissue (La Morgia et al., 2013; Yu et al., 2014) (Table 1).

In the normal retina of vertebrates, α-syn is expressed at photoreceptor axon terminals of vertebrates, as well as in several subtypes of bipolar and amacrine retinal cells. This protein is present in presynaptic, but not postsynaptic, terminals of retinal neurons in both IPL and OPL, where it could be associated with synaptic vesicles to modulate neurotransmission (Martinez-Navarrete et al., 2007). However, α-syn aggregates are related to neurodegenerative disorders, including PD. In postmortem PD eyes, α-syn aggregates have been observed inside the neurons of different retinal layers, including the border of the INL, the IPL, and the GCL. These locations suggest a substrate for the visual impairment in PD (Bodis-Wollner et al., 2014). Maurage et al. also reported the presence of α-syn inclusions in the OPL and a lower cone density in a patient suffering dementia with Lewy bodies (Maurage et al., 2003). Additionally, in transgenic mice overexpressing α-syn, an accumulation of this protein has been found in the INL, GCL, and NFL (Surguchov et al., 2001; Figure 1B, Table 1).

In PD, in addition to substantia nigra dopaminergic neuron degeneration, the DA content in the retina diminishes. This deficiency could alter visual processing by altering the ganglion cells receptive fields (Djamgoz et al., 1997). Retinas having a dopaminergic deficiency associated with the loss of amacrine cells, which provide input to the ganglion cells, can lose RGCs. This fact is has been observed both in human PD and in PD model in monkeys treated with 1-methyl-4-phenyl-1,2,3,6-tetrahydropyridine (MPTP) (selective neurotoxin which destroys DA neurons) (Bodis-Wollner, 1990; Tatton et al., 1990; Cuenca et al., 2005). This loss can be mediated by the impoverished dopaminergic input, which contributes to an alteration in the glutamate production and the atrophy of inferotemporal circumpapillary RNFL in PD patients (Inzelberg et al., 2004). These data agree with the observations in PD patients, in which a thinning of inner retinal layer (15–20%) has been demonstrated in the macular region. This percentage of thinning does not necessarily cause a vision loss (Hajee et al., 2009; Figure 1B, Table 1). Also in the retina of mice treated with rotenone (pesticide that elicits DA neuron degenerations), an experimental model of PD, a correlation between functional and structural alterations were located in the retina, specifically in the photoreceptors and their synaptic connections with second-order neurons (Esteve-Rudd et al., 2011).

Very few studies analyze retinal glial cells in PD. In a transgenic mouse model overexpressing α-syn, an accumulation of α-syn has been found in glial cells of the INL (Surguchov et al., 2001). In a PD model with the administration of MPTP increased GFAP immunostaining, glutamine synthetase (Müller cell marker), and CD11b (microglial marker) were detected, indicating an activation of retinal glial cells (Chen et al., 2003). In the same experimental model, Nagel et al. also observed astrogliosis in retinal tissue, without changes in the number of tyrosine hydroxylase (TH)+ amacrine cells, postulating that other retinal neurons can be affected, even non-neuronal cells (Nagel et al., 2009). In addition, a non-proliferative gliosis of GFAP+ Müller cells was found in the MPTP model of PD. This gliosis was accompanied of milder declines in TH+ amacrine cells, followed by stronger recoveries without neurogenesis (Cho et al., 2012). Müller cells constitute the main glial cell type in the retina where it interacts with virtually all cells displaying functions relevant to retinal physiology. Müller cells are able to synthesize and release DA to the extracellular medium. Thus, the dopaminergic Müller cells can be used as a source of DA in cell-therapy procedures (Stutz et al., 2014; Table 1).

### Glaucoma

Glaucoma, the second leading cause of blindness in the world, is characterized by the irreversible RGC loss, leading to a vision loss (Quigley and Broman, 2006). In the early stages of the disease, the reactivation of the glial cells leads to the progression of glaucomatous damage (Tezel, 2009). As mentioned above, when neurons are damaged, microglial cells respond by adopting an activated phenotype (Kreutzberg, 1995; Graeber and Streit, 2010). In glaucoma, activated microglia can exhibit morphological changes, proliferate, migrate, or can change the expression of different enzymes, receptors, growth factors, and cytokines (Rojas et al., 2014). An overexpression of these latter inflammatory mediators can contribute to retinal degeneration (Langmann, 2007; Karlstetter et al., 2010). Also, microglia can act as antigen-presenting cells and even transform into phagocytes (Luo et al., 2010; Kettenmann et al., 2011; Karlstetter et al., 2015; Ransohoff and El Khoury, 2015). Unfortunately the role of the microglia in the pathophysiology of glaucoma is poorly understood, and thus better knowledge of the function of microglial cells in this disease is necessary.

As mentioned above, microglial activation is one of the first events in glaucomatous neurodegeneration (Williams P. A. et al., 2017), but even this activation is prior to the RGC loss (Ebneter et al., 2010; Bosco et al., 2011). In experimental glaucoma models, it has been observed that after treatment with minocycline (Bosco et al., 2008) or with a high dose of irradiation (Bosco et al., 2012), there was a reduction of microglial activation and thus lower RGC death. In addition, in DBA-2J mice a significant quantitative correlation has been established between the microgliosis and the axon loss in the optic nerve (Bosco et al., 2016; Figure 1C, Table 1).

Neurons can induce an inflammatory response in microglial cells after an injury. Nucleotides released by damaged neurons can up-regulate the purinergic receptors of the microglia, activating their phagocytic ability, motility, and migration (Koizumi et al., 2007; Ohsawa et al., 2007; Wu et al., 2007). It has been demonstrated, in an experimental mouse glaucoma model, that deficiencies in the activation of CX3R1 increase microglial activity, neurotoxicity, and the RGC death (Wang et al., 2014). In addition, in the experimental model of glaucoma, there is an early change in the CD200R/CD200 expression which regulates the microglial activity and precedes RGC death (Taylor et al., 2011). The damaged neurons can release head-shock proteins (HSP), triggering the oxidative response in the microglial cells. These proteins can activate the innate immune system via TRLs in the glaucoma (Tezel, 2009; Karlstetter et al., 2015). In the human glaucoma, high levels of HSP27, HSP60, HSP7, and antibodies against HSPs (Cagnin et al., 2001; Streit et al., 2004; Wyss-Coray, 2006; Stewart et al., 2010; Parpura et al., 2012; Calsolaro and Edison, 2016; De Strooper and Karran, 2016; Bennett et al., 2017) have been found. Furthermore, the dying neurons release the protein HMGB1, which binds to the CD11b receptor of the microglia to induce the production of inflammatory and neurotoxic factors. In experimental glaucoma the elimination of the CD11b receptor has a neuroprotective role since it prevents the microglial activation (Nakazawa et al., 2006).

In glaucoma patients, an overexpression of γ–synuclein has been demonstrated in ganglion cell axons as well as in glial cells of the lamina and postlamina cribosa of the optic nerve. Synuclein has an important role in neurodegenerative diseases, and these findings suggest possible synuclein involvement in glaucomatous alterations in the optic nerve (Surgucheva et al., 2002).

In experimental glaucoma and in the DBA/2j spontaneous mouse glaucoma model, amyloid precursor protein and Aβ were found in the RGCs (Figure 1C) in relation to increased IOP (McKinnon et al., 2002; McKinnon, 2003; Goldblum et al., 2007). In addition, abnormal tau (AT8) and phosphorylated tau were found to be present in human ocular tissues of uncontolled IOP and in donor eyes with glaucoma (Gupta et al., 2008; Ning et al., 2008). This implies that Aβ accumulation in the retina is involved in the pathogenesis of glaucoma, this Aβ deposition being related to microglial activation and neuroinflammation (Bolos et al., 2017; Figure 1C, Table 1).

When microglia are activated, they can adopt different morphologies. In experimental models of glaucoma, activated microglia acquire several morphological phenotypes: stellate cells with thick processes, hyper-ramified cells, rounded cells, amoeboid cells (which act as macrophages, phagocytizing cellular debris) and rod-like microglia. The rod-like microglia are related to neurodegeneration, in the experimental glaucoma model, and the presence of this cell type is restricted to eyes with neuronal damage. It seems that the rod-like microglia might be involved in the active removal or “stripping” of the synaptic contacts (Gallego et al., 2012; de Hoz et al., 2013; Rojas et al., 2014).

In addition to the different morphologies, activated microglia can adopt different functional phenotypes in response to neuronal damage. After injury, the cytokines released by the damaged cells (e.g., IFN-γ) give rise to the microglial activation, acquiring a M1-like phenotype. This phenotype is characterized by production of proteolytic enzymes and pro-inflammatory cytokines (TNF-α, IL-1β, IL-6, IL-12, and NO) promoting tissue inflammation (Varnum and Ikezu, 2012; Gonzalez et al., 2014; Jones and Bouvier, 2014). In human glaucoma and in the experimental models of glaucoma, high levels of these pro-inflammatory cytokines have been found (Neufeld et al., 1997; Shareef et al., 1999; Tezel et al., 2001; Nakazawa et al., 2006; Vidal et al., 2006; Lee et al., 2014; Madeira et al., 2015).

In experimental glaucoma, it has been observed that the activated microglia can migrate to remove the damaged or dead cells (Bosco et al., 2012; Rojas et al., 2014). In the human glaucoma the amoeboid microglia are located in the lamina cribrosa phagocyting the damaged axons (Neufeld, 1999). The morphology change of the microglia from the ramified shape to the amoeboid phagocytic shape is associated with the expression of different surface markers such as: MHC-II (OX6), CD68, Griffonia simplicifolia isolectin B4, complement receptor 3 (CD11b/CD18, OX42), and F4/80 (Kreutzberg, 1996; Streit et al., 1999). In a unilateral experimental glaucoma model, CD68 expression (a member of the scavenger-receptor family) was observed in the retinal microglia (Rojas et al., 2014). The migration and the proliferation of the microglial cells are regulated by soluble factors or by the extracellular matrix changes of damaged CNS tissues. It has been found that microglia of the optic-nerve head express different matrix metalloproteinases and their inhibitors, indicating their participation in the remodeling of the extracellular matrix (Yuan and Neufeld, 2001; Garden and Möller, 2006).

The activation of the microglia also involves higher numbers of microglial cells. This fact it has been observed in human glaucoma and in glaucoma animal models (Giulian and Ingeman, 1988; Yuan and Neufeld, 2001; Naskar et al., 2002; Inman and Horner, 2007; Johnson et al., 2007; Gallego et al., 2012; de Hoz et al., 2013; Rojas et al., 2014). The microglia mitosis can be stimulated by neurotrophic factors (BDNF, NT-3) and several cytokines (macrophage colony-stimulating factors, granulocyte macrophage CSF, IL-1β, IL-4, and IFN-γ) (Garden and Möller, 2006).

In the classical M1 activation, the MHC II, CD86, and Fcγ receptors are up-regulated, because this phenotype is oriented to antigen presentation and the killing of intracellular pathogens (Taylor et al., 2005; Cherry et al., 2014). Under physiological conditions, some microglial cells express very low levels of MHC-II, although certain pro-inflammatory cytokines (e.g., TNF-α or IFN-γ) can upregulate MHC-II expression by microglial cells. In this context, both for glaucoma patients (Yang et al., 2001b; Tezel, 2009; Ebneter et al., 2010) and in animal models of glaucoma (Ebneter et al., 2010; Gallego et al., 2012; de Hoz et al., 2013; Rojas et al., 2014) there is evidence for increased expression of MHC-II molecules in glial cells. In a glaucoma model, after 15 days of ocular hypertension (OHT), most of microglial cells were MHC-II + while the CD86 expression was observed only in some amoeboid and rounded Iba-1+cells in the NFL and the GCL (Rojas et al., 2014). The fact that most of microglial cells were CD86- could prevent T-cell activation by their omission of co-stimulation, leading to a downregulation of the immune response (Broderick et al., 2000). In addition, in an experimental glaucoma model the MHC-II upregulation by the activated microglia in the optic nerve could be associated with more severe RGC degeneration (Chidlow et al., 2016). It has been observed in an experimental glaucoma model that caffeine administration decreases the microglia MHC-II upregulation reducing microglial activation and increasing RGC survival (Madeira et al., 2016).

After M1 activation, the microglial cells can return to a state of rest, adopting a transitory state of M2 activation. In this state, the microglia can upregulate CD68, CD206, and Ym1 (Menzies et al., 2010; Komori et al., 2011; Varnum and Ikezu, 2012; Jones and Bouvier, 2014; Zhou et al., 2014). In an experimental model of unilateral glaucoma, it was observed that the only cells expressing Ym1 were amoeboid Iba-1 + cells in the NFL and GCL of the OHT retinas. The authors postulated that most of the microglial cells in this OHT model were serving functions not related with the M2 microglial phenotype (Rojas et al., 2014).

In glaucomatous eyes, the chronic stress in the tissue can induce the rupture of the blood-retinal barrier, allowing the contact of nervous tissue of the retina and the optic nerve with systemic immune cells (Tezel, 2009). In addition, chemokines (CCL2, CCL5, CCL20, CXCL10, CXCL12, CXCL1, CXCL2, and CX3CL1) released by reactive astrocytes can recruit dendritic cells, microglia, monocytes/macrophages, and T-cells into the inflamed tissue (Farina et al., 2007; Gonzalez et al., 2014). In a chronic glaucoma model DBA/2J, the loss of CX3CL1 signaling increased the infiltration of peripheral macrophages (Breen et al., 2016). The role of monocytes in the survival of RGCs is controversial. In an experimental model of OHT it was observed that an increased number of monocytes could be protective (London et al., 2011). However, in a genetic model of glaucoma (DBA/2J) the irradiation that lowered the number of monocytes boosted RGC survival (Howell et al., 2012).

In addition, the blood retinal barrier breakdown (Figure 1C, Table 1) can allow the entry of complement proteins, thus activating the complement in the retinal tissue (Karlstetter et al., 2015). For retinal homeostasis, the level of complement proteins should be low. However, the complement constituents can be activated by inflammatory cytokines (e.g., TNF-α, INF-γ, and IL-6) produced under inflammatory conditions such as glaucoma (Karlstetter et al., 2015). In the retina of the glaucomatous eyes an upregulation of the component complement C1q has been observed (Steele et al., 2005; Stasi et al., 2006). Microglial cells respond to C1q upregulation by eliminating the targeted synapses (Steele et al., 2005; Stasi et al., 2006). Thus, the involvement of the immune system in glaucomatous pathology has been postulated. Recently, it has been suggested that oral microbiome could be related to glaucoma pathophysiology, through microglial activation mediated through TLR4 signaling and complement upregulation (Astafurov et al., 2014). Apart from the chronic activation of resident immunoregulatory glial cells, the presence of plasma cells in the retina, and the complement activation (Tezel, 2009), high levels of autoantibodies and deposition of immunoglobulins have been found in the glaucomatous neurodegeneration (Wax et al., 1998; Hammam et al., 2008; Gramlich et al., 2013; Pinazo-Duran et al., 2013). It has even been speculated that the glaucoma would be mediated by an autoimmune mechanism and that both innate and adaptive responses accompany this pathology (Tezel, 2009, 2013). The serum of glaucoma patients has been found to contain high levels of antibodies (e.g., against HSPs; Maruyama et al., 2000; Wax et al., 2001; Tezel et al., 2004; Grus et al., 2008). Moreover, serum alteration of the populations of T-cell repertoires and of interleukin-2 receptors has been detected (Yang et al., 2001a). In view of the evidence mentioned above, the immune response could be involved in the pathogenesis of the glaucoma.

## Conclusion

AD, PD, and glaucoma are neurodegenerative diseases that share a common pathogenic mechanism, in which the neuroinflammation, in the form of microglial activation, plays an important part. The differential activation of microglia (M1 or M2 phenotypes) can produce a neurotoxic or neuroprotective environment, and could constitute a key in neuroinflammation regulation. In the search for a new strategy to control neuroinflammation, it might be more effective to change the M1 phenotype to the M2 phenotype than to block microglial activation completely. In the regulation of microglial activation, several cell types including, neurons, astrocytes, and T-cells are involved. When the neuroinflammatory process is triggered by protein aggregates (Aß, α-syn, pTau etc.), peripheral immune cells infiltrate CNS and prompt more activation on resident microglia, favoring neuroinflammatory processes.

Neuroinflammatory processes occur not only in the brain but also in the retina, because the retina is a projection of the CNS. Thus AD, PD, and glaucoma share neuroinflammatory changes in the retinal tissue. The follow up of neuroinflammatory processes in the retinal tissue may be useful for the early diagnosis and monitoring of neurodegenerative diseases. Future research could therefore address these issues to provide fuller knowledge of neuroinflammatory events that occur in AD, PD, and glaucoma, especially the contribution of microglia. This might help in the development of new therapeutic strategies to control neuroinflammation and thereby spur progress in treating these neurodegenerative diseases.

## Author contributions

Conception of the work: Rd, JS, AR, ES, BR, AT, and JR. Acquisition, analysis and interpretation of data for the work: Rd, JS, AR, ES, and JR. Bibliographic research: Rd, JS, AR, ES, DA, PR, IL, and JR. Drafting the work: Rd, JS, AR, ES, DA, IL, and JR. Revising critically for important intellectual content: Rd, JS, AR, ES, DA, IL, PR, BR, AT, and JR. Final approval of the version to be published Rd, JS, AR, ES, IL, DA, PR, BR, AT, and JR. Agreement to be accountable for all aspects of the work in ensuring that questions related to the accuracy or integrity of any part of the work are appropriately investigated and resolved: Rd, JS, AR, ES, DA, IL, PR, BR, AT, and JR.

### Conflict of interest statement

The authors declare that the research was conducted in the absence of any commercial or financial relationships that could be construed as a potential conflict of interest.
